# Polymorphisms in the 5-HTR2A gene related to obstructive sleep apnea syndrome

**DOI:** 10.1590/S1808-86942011000300013

**Published:** 2015-10-19

**Authors:** Vânia Belintani Piatto, Thiago Bittencourt Ottoni de Carvalho, Nely Silva Aragão de Marchi, Fernando Drimel Molina, José Victor Maniglia

**Affiliations:** 1 Doctoral degree in the Health Sciences, adjunct professor III-D at the Otorhinolaryngology and Head & Neck Surgery Department, FAMERP; 2 Medical graduate, fourth year resident in otorhinolaryngology and head & neck surgery; 3 Doctoral degree in the Health Sciences. Head of the Sleep Ambulatory Unit, FAMERP/FUNFARME; 4 Doctoral degree in the Health Sciences. Head of the Otorhinolaryngology and Head & Neck Surgery Outpatient Unit, FAMERP/FUNFARME; 5 Associate professor at the Otorhinolaryngology and Head & Neck Surgery Department, FAMERP; São José do Rio Preto Medical School (Faculdade de Medicina de São José do Rio Preto, SP - FAMERP) - Av. Brigadeiro Faria Lima 5416 São José do Rio Preto SP 15090-000

**Keywords:** polymorphism genetic, receptor serotonin 5-ht2a, serotonin, sleep apnea obstructive

## Abstract

Obstructive sleep apnea syndrome (OSAS) is one of the most complex disorders of sleep; it involves several genetic factors that contribute to the phenotype. Serotonin (5-HT) regulates a variety of visceral and physiological functions, including sleep. Gene 5-HTR2A polymorphisms may change the transcription of several receptors in the serotoninergic system, thereby contributing to OSAS.

**Aim:**

To investigate the prevalence of T102C and -1438G/A polymorphisms in the 5-HTR2A gene of patients with and without OSAS.

**Material and Method:**

A molecular study of 100 index-cases and 100 controls of both genders. DNA was extracted from blood leukocytes samples and the regions that enclose both polymorphisms were amplified with PCR-RFLP.

**Study design:**

A cross-sectional case study.

**Results:**

There was a significant prevalence of males in index cases compared to controls (p<0.0001). No significant genotypic differences between cases and controls were found in T102C polymorphisms (*p*=1.000). There were significant differences between the AA genotype of -1438G/A polymorphisms and patients with OSAS (OR:2.3; CI95%:1.20-4.38, *p*=0.01).

**Conclusion:**

Serotonergic mechanisms may be related to OSAS. There were no differences in the prevalence of T102C polymorphisms in patients with OSAS and the control group. There is evidence of an association between the -1438G/A polymorphism and OSAS.

## INTRODUCTION

The obstructive sleep apnea syndrome (OSAS) is a common disorder of sleep affecting from 2% to 4% of adult males. It may be characterized by recurring sleep-induced pharyngeal airway collapse resulting in hypoxemia and hypercapnia.^1^ Several genetic factors are probably involved, since there are also many phenotypic components.^2^, ^3^, ^4^, ^5^

Changes in central nervous control of upper airway muscles are considered an important component of this syndrome.^6^ The genioglossus muscle is innervated by neurons that originate in the hypoglossal nucleus within the brainstem; contractions of this muscle during inspiration help ventilate the lungs and keep upper airways open during wakefulness and sleep.^7^, ^8^ Groups of serotonergic and noradrenergic cells may generate parallel patterns by stimulating directly the motor cells of the hypoglossal nerve, thereby regulating these dilating muscles. Thus, activation of these cell groups increases the activity of the genioglossus muscle.^7^, ^8^, ^9^

It is evident that serotonin (5-hydroxytryptamine or 5-HT), a central nervous system neurotransmitter, is involved in regulating several visceral and physiologic functions such as sleep, appetite, thermoregulation, pain perception, hormone secretion, and sexual behavior. Changes in the serotonergic system have been implied in several human diseases such as depression, headaches, epilepsy, obsessive-compulsive behaviors, temporomandibular dysfunction, and affective disorders.^6^, ^7^, ^8^, ^9^

Serotonin also has a relevant role in upper airway patency, as it excites airway motor neurons and intrinsically excites the motor neurons in the brainstem. The activity of neurons that provide 5-HT to motor neurons declines with sleep; most of the functional studies have shown that 5-HT and serotonergic neurons exert a significant excitatory effect on respiratory motor neurons in vivo and in vitro. The activity of these neurons is maximal during wakefulness, and minimal during REM sleep. The sleep-related behavior of 5-HT on motor neurons results in upper airway collapse and the ensuing respiratory obstruction.^10^, ^11^

Serotonin operates by means of a large family of 5-HT receptors; these include the 5-HT2 receptors that comprise three subtypes (5-HT2A, 5-HT2B, and 5-HT2C) that have a similar molecular structure, pharmacology, and transduction signal patterns. The 5-HT2A and 5-HT2C subtypes have an important role in keeping upper airways stable and maintaining normal breathing in obese subjects.^12^ One of the receptor subtypes for serotonin - 5-HT2A - is mostly excitatory for the motor neurons of the hypoglossal muscle; the 5-HT2C subtype is excitatory for neurons in several areas of the brain. Although few excitatory responses have been analyzed with selective agents for receptor subtypes, evidence suggests that in some cases 5-HT2A mediates the responses, while in others, 5-HT2C is involved.^12^

The genes in the family 5-HT2 receptors (named HTR2 genes) have two introns (for genes *5-HTR2A* and *5-HTR2B)* or three (*5-HTR2C* gene) in the coding sequence, which are all coupled positively to phospholipase C, and which mobilize intracellular Ca^2^+.^12^ The *5-HTR2A* gene is on chromosome 13 (13q14-q21); it has a relatively high amino acid sequence, identical to *5-HTR2C*, although lower compared to the *5-HTR2B* gene. The *5-HTR2C* gene is located on chromosome X (Xq24).^12^

A functional promoting variant of the *5-HTR2A* gene may differentially alter transcription, thereby changing the number of receptors. Polymorphisms in the *5-HTR2* gene are associated with several diseases, including the OSAS; it affects the serotonergic system, thereby contributing to airway collapse during sleep. The excitatory effects of the 5-HT2C receptor are small; although the *5-HTR2C* gene polymorphism is functional, the effect of an active polymorphic allele may not be sufficient for this polymorphism to be associated with the apnea/hypopnea index (AHI) because this subtype of receptor is less dominant in the nucleus of the hyplglossus.^13^, ^14^ Conversely, a *5-HTR2A* gene polymorphism has recently been found in patients with OSAS: it is defined by a T->C substitution in the nucleotide position 102^8^, and a G->A substitution in position -1438 of the promoting region of the gene.^9^

## OBJETIVE

The purpose of this study was to investigate the prevalence of T102C and -1438G/A polymorphisms in the *5-HTR2A* gene with the polymerase chain reaction/restriction fragment length polymorphism (PCR/RFLP) test in a sample of patients with and without the OSAS.

## MATERIAL AND METHOD

The institutional review board approved this study (Opinion no. 342/2006) based on the regulating norms for research on human beings - Resolution 196/96 of the Ministry of Health.

A cross-sectional cohort study was carried out from 1 July 2008 to 30 June 2010 of 100 index cases with the OSAS. There were 73 male and 27 female subjects with ages ranging from 23 to 70 years. The control group comprised 100 patients (40 male and 60 female) aged from 17 to 70 years. A full medical history was taken to investigate the presence of agitated sleep, night apnea, daytime drowsiness, medication use, arterial hypertension, and depression. A systemic and otorhinolaryngological physical examination was carried out to assess the body mass index (BMI), the neck diameter, neck tumors, craniofacial malformations, and the nasal and oral cavities. Subjects then underwent general and specific tests for OSAS - cephalometry, nasofibroscopy with the Müeller maneuver, and polysomnography.

The following inclusion criteria were applied:

a) meeting criteria A or B, plus C for the diagnosis of the syndrome.
A excessive daytime drowsiness not explained by other factors;
B two or more of the following findings not explained by other factors:
  gasping during sleep;
  recurring awakenings from sleep;
  restless sleep, daytime fatigue;
  poor concentration.
C nighttime monitoring demonstrating more than five episodes of respiratory obstruction per hour during sleep. These events may include any combination of obstructive apnea/hypopnea or respiratory efforts with awakening.
  b) absence of other somatic or laboratory findings in the general physical examination and tests;
  c) absence of craniofacial dimorphism or temporomandibular conditions, as seen in the specific physical examination;
  d) absence of drug dependency, alcoholism, depression, or dementia, as investigated in the clinical history and psychiatric assessments;
  e) absence of apparent genetic syndromes, as investigated by the clinical/genetic physical examination;
  f) presence or absence of other cases in the family;
  g) maximum age - 70 years.
  h) BMI ≤ 35.
  Exclusion criteria:
  a) Patients aged over 70 years, as the prevalence of central sleep apnea increases with age;
  b) presence of psychiatric disorders;
  c) altered laboratory tests, and findings on the general and specific physical examination;
  d) not presenting the minimal criteria for diagnosing the syndrome;
  e) BMI > 35.

Patients for the control group were selected based on the same criteria above for patients with the OSAS, except for the AHI/hour, which for this group was ≤ 5.

The following data were gathered: age at the moment polysomnography was done, the BMI, and the AHI from the recordings of the polysomnography equipment (Stellat System QC, Harmonie TM, Canada). Patients were allocated to the following age groups: adolescent (11 to 17 years), young adult (18 to 40 years), adult (41 to 65 years), and elderly (> 65 years).^15^, ^16^ Patients were grouped according to the following classification based on the AHI: mild obstructive sleep apnea hypopnea syndrome (OSAHS) - AHI = 5 to 15.9 events/hour), moderate OSAHS - AHI = 16 to 30 events/hour), severe OSAHS - AHI > 30 events/hour).^17^ Patients were also grouped according to the BMI based on the WHO classification: ideal weight (18.5-24.9 kg/m^2^), overweight (25.0-29.9 kg/m^2^), obesity grade I (30.0-34.9 kg/m^2^), and obesity grade II (35.0-39.9 kg/m^2^).^18^ A BMI ≤ 35 limit was established to avoid effects of higher obesity grades on the AHI.

### Molecular study

A total blood sample (4.0 mL) was collected in a Vacutainer® tube with anticoagulant (EDTA), after consent was obtained from patients or caretakers. Genomic DNA was extracted from blood samples using the Illustra Blood GenomicPrep Mini Spin Kit (GE Healthcare)™ according to the manufacturer's instructions.

Nuclear DNA fragments including the polymorphic region of the *5-HTR2A* gene were amplified by the polymerase chain reaction (PCR) using the FideliTaq™ PCR MasterMix (2X) (GE Healthcare®) kit to detect both polymorphisms (T102C and -1438G/A). In this reaction two pairs of primers - synthetic oligonucleotides - were used; the oligonucleotide sequence in the primers and the PCR conditions were taken from the literature.^8^, ^9^

A 342 pb fragment was amplified as product of the PCR for the T102C polymorphism; it was then submitted to the restriction analysis (restriction fragment length polymorphism or RFLP) using the MspI enzyme (New England Biolabs)™ for 2:30 h at 37°C.^8^ Digestion of the mutant homozygotic sample fragment (CC) for the T102C polymorphism (both mutant alleles) yields two fragments (217 pb and 125 pb) because of recognition of the Mspl enzyme restriction site by substitution of the T->C nitrogenated bases in the position 102 of the *5-HTR2A* gene. The heterozygote samples (TC) for this polymorphism, after enzyme digestion, yielded three fragments: 342 pb (wild allele-T), 217, and 125 pb (mutant allele-C). Samples with no polymorphism, therefore normal homozygotes (TT), contain only a 342 pb fragment, as the enzyme site is not recognized.

Similarly, to detect the -1438G/A polymorphism, a 469 pb fragment was obtained following PCR; it was subjected to RFLP with the MspI enzyme (New England Biolabs)™ for 2:30 h at 37°C.^9^ Digestion of a fragment from normal homozygotic samples (GG) for the -1438G/A polymorphism yields two fragments (243 pb and 226 pb) because of recognition of the Mspl enzyme restriction site. The mutant homozygotic samples (AA) for this polymorphism contain a single 469 pb fragment, as the enzyme site is not recognized by substitution of the G->A nitrogenated bases in the position -1438 of the *5-HTR2A* gene; the heterozygotic samples (GA) contain three fragments: 469 pb (mutant allele-A), 243, and 226 pb (wild allele-G).

The products of both reactions (PCR/RFLP) were analyzed using 2% agarose gel electrophoresis in a TBE 1X buffer containing ethidium bromide at 0.5 mg/mL, under ultraviolet light, to confirm the success of the reaction; the gel was photographically documented.

### Statistics

Descriptive statistics was applied to establish normalcy of results. Student's t test (two-tailed) was applied for independent samples with a normal distribution; the Mann-Whitney test was used for samples with a non-normal distribution. If applicable, the chi-square test was used for comparisons among variables, and the odds ratio (95% confidence interval). The significance level was 5%. The Hardy-Weinberg equilibrium analysis was made for the genotype distribution.

The statistical tests were done using the GraphPad InStat version 3.00 software (GraphPad Software Inc, San Diego California USA; www.graphpad.com).

## RESULTS

Of 100 patients with apnea (index cases), 73 (73%) were male and 27 (27%) were female. The age ranged from 23 to 70 years in males (mean - 50.1 years; SD ± 12.5) and 43 to 65 years in females (mean - years; SD ± 6.0). The BMI ranged from 21.2 to 35 kg/m^2^ in males (mean - 29.5 kg/m^2^; SD ± 3.8), and 21 to 35 kg/m^2^ in females (mean - 28.6 kg/m^2^; SD ± 3.5). The AHI ranged from 5.8 to 115 in males (mean - 37.5; SD ± 26.3) 5.4 to 79 in females (mean - 19.6; SD ± 19.3).

Of 100 patients in the control group, 40 (40%) were male and 60 (60%) were female. The age ranged from 17 to 66 years in males (mean - 46.1 years; SD ± 12.3) and 21 to 70 years in females (mean - 43.6 years; SD ± 11.7). The BMI ranged from 20 to 34.6 kg/m^2^ in males (mean - 26.8 kg/m^2^; SD ± 3.9) and 20 to 35 kg/m^2^ in females (mean - 27.1 kg/m^2^; SD ± 4.0). The AHI ranged from 0 to 4.9 in males (mean - 2.3; SD ± 1.5) and 0 to 4.9 in females (mean - 1.6; SD ± 1.4).

Table 1 presents demographic data for the 100 index cases and 100 controls; Table 2 presents these data as mans ± standard deviation.

**Table 1:** Distribution of demographic data of index cases (n=100) compared to the control group (n=100).

Variables	Index-cases n (%)	Controls n (%)	p*
Gender Male	73 (73)	40 (40)	<0.0001
Female	27 (27)	60 (60)	
BMI Ideal weight	13 (13)	32 (32)	
Overweight	44 (44)	41 (41)	0.0081
Obesity grade I	38 (38)	24 (24)	
Obesity grade II	05 (05)	03 (03)	
Age Adolescent	0 (0)	01 (01)	
Young adult	12 (12)	35 (35)	0.0007
Adult	80 (80)	61 (61)	
Elderly	08 (08)	03 (03)	
	Data gathered during polysomnography n (%)		
AHI			
Normal	--	100 (100)	
Mild	40 (40)	--	NA
Moderate	15 (15)	--	
Severe	45 (45)	--	

BMI - body mass index.

AHI - apnea/hypopnea index.

NA - Not analyzed.

* Chi-square test.

**Table 2:** Clinical and polysomnographic parameters of index cases compared to controls.

Parameters	Index-cases (n=100)	Controls (n=100)	*p*
BMI (grade)	29.3 ± 3.7	27.0 ± 3.9	p<0.0001*
Age (years)	50.6 ± 11.1	44.6 ± 12.0	p=0.0004+
AHI	32.6 ± 25.7	1.9 ± 1.5	p<0.0001+

Values are presented as the mean ± standard deviation.

BMI - body mass index.

AHI - apnea hypopnea index.

* Student's t test (two-tailed) for independent samples.

+ Mann-Whitney test.

Males predominated among index cases (73%) and females predominated in the control group (60%), which was significant (*p*< 0.0001).

The group of patients with OSAS had a higher prevalence of overweight and obesity grade I in the BMI classification -82% of cases; in the control group, ideal weight and obesity grade I were more prevalent (73%). This difference between groups was significant (*p*=0.0081).

The highest age prevalence in both groups (index cases and controls) was in the young adult (92%) and adult 96% ranges. There were no adolescents among the index cases. The statistical analysis of this variable in index cases and controls showed that it was significant (*p*<0.0001).

Table 2 presents the mean BMI values, the age, and the AHI, which were significantly higher among the index cases compared to the mean values in the control group.

Table 3, Table 4, Table 5, Table 6, Table 7 present the molecular results and the comparison among variables.

**Table 3:** Distribution of genotypes in index cases and controls for T102C and -1438G/A polymorphisms of the 5-HTR2A gene.

Genotype	Index-cases n (%)	Controls n (%)	p*
TT	23 (23)	20 (20)	0.8711
TC	66 (66)	69 (69)	
CC	11 (11)	11 (11)	
GG	4 (4)	10 (10)	0.0177
GA	61 (61)	71 (71)	
AA	35 (35)	19 (19)	

* Chi-square test.

**Table 4:** Genotype relation between index cases and controls for T102C and -1438G/A polymorphisms of the 5-HTR2A gene.

Genótipo (Casos × Controles)	*Odds ratio* (OR)	IC 95%	*Chi-square*	^*P*^
TT and TC (89 × 89)	1.0	0.41-2.43	0.000	1.000
CC (11 × 11)	1.0	0.41-2.43		
GG and GA (65 × 81)	0.44	0.23-0.83	5.71	0.0169
AA (35 × 19)	2.3	1.20-4.38		

CI - 95% confidence interval

**Table 5:** Gender distribution of index cases and controls relative to the genotypes for T102C and -1438G/A polymorphisms of the 5-HTR2A gene.

Genotype	Index-cases		Controls		P*
	Male n (%)	Female n (%)	Male n (%)	Female n (%)	
TT and TC	64	25	33	56	0,3387
CC	9	2	7	4	
GG and GA	40	25	31	50	<0,0001
AA	33	2	9	10	

* Chi-square test.

**Table 6:** Distribution of the BMI (body mass index) of index cases and controls relative to the genotypes for T102C and -1438G/A polymorphisms of the 5-HTR2A gene.

Genotype	Index-cases n (%)		Controls n (%)		P*
	20 ≤ BMI < 25 Kg/m^2^	25 ≤ BMI ≤ 35 Kg/m^2^	20 ≤ BMI < 25 Kg/m^2^	25 ≤ BMI ≤ 35 Kg/m^2^	
TT and TC	12 (12)	77 (77)	27 (27)	62 (62)	0,7542
CC	1 (1)	10 (10)	5 (5)	6 (6)	
GG and GA	6 (6)	59 (59)	24 (24)	57 (57)	0,0182
AA	7 (7)	28 (28)	8 (8)	11 (11)	

* Chi-square test.

**Table 7:** Distribution of the AHI (apnea/hypopnea index) of index cases relative to the genotypes for T102C and -1438G/A polymorphisms of the 5-HTR2A gene.

Genotype	Index-cases n (%)		P*
	5 ≤ AHI ≤ 30	AHI > 30	
TT and TC	50 (50)	39 (39)	0,7238
CC	5 (5)	6 (6)	
GG and GA	38 (38)	27 (27)	0,4608
AA	17 (17)	18 (18)	

Chi-square test.

The T and C allele frequencies for the index cases were T=0.56 and C=0.44; among controls they were T=0.545 and C=0.455. This difference was not statistically significant (*p*=0.8406). The G and A allele frequencies for the index cases were G=0.345 and A=0.655; among controls they were G=0.455 and A=0.545. This difference was statistically significant (*p*=0.0321).

There was a statistical difference in genotypes of the -1438G/A polymorphism among index cases compared to the control group (*p*=0.0177).

The genotype frequencies for the T102C polymorphism were not in Hardy-Weinberg equilibrium both among index cases (χ^2^=11.511; *p*=0.00069) and controls (χ^2^=15.309; *p*=0.00009). The genotype frequencies for the -1438G/A polymorphism were not in Hardy-Weinberg equilibrium both among index cases (χ^2^=12.229; *p*=0.00047) and controls (*χ* ^2^=18.628; *p*=0.00002).

Table 4 presents as odds ratio the relation of genotypes in index cases and controls.

There was no statistical difference between genotypes TT and TC among cases × controls; the same applied to the genotype CC, as both groups had the same frequency of these genotypes.

Although the frequency of genotypes GG and GA was high in both groups, the control group had a significantly higher frequency compared to index cases. The frequency of the AA genotype was significantly higher among index cases compared to controls.

The prevalence of the -1438G/A polymorphism genotypes among male index cases was significant compared to controls (*p*<0.0001).

There was a statistically significant association between -1438G/A polymorphism genotypes and a BMI from 25 to 35 kg/m^2^ among index cases compared to controls (*p*<0.0182).

There was no association between both polymorphisms and the severity of OSAS among index cases.

## DISCUSSION

The serotonergic system is an important component of sleep and relaxation of airways during sleep. Thus, factors that affect this system may result in sleep and respiratory disorders. The 5-HT2A receptor is an essential component of the serotonergic system; it is under genetic control. Gene polymorphisms that code for the receptor may alter the functional state and serotonergic activity.^9^, ^12^ Thus, our study aimed to investigate the prevalence of T102C and -1438G/A polymorphisms of the *5-HTR2A* gene in patients with and without OSAS. Until this time there were no published Brazilian papers on this topic; only two studies in the literature had reported the frequency of these polymorphisms and their association with OSAS,^8^, ^9^ which underlines the importance of the present study.

All subjects underwent polysomnography to obtain the AHI, through which the diagnosis of OSAS could be made to compose the groups (OSAS and controls). This criterion was followed strictly so that no patient with only a clinical exclusion diagnosis of OSAS is included in the control group, as was done in other published studies.^6^, ^8^, ^9^

The OSAS is more frequent in men of all age groups, although the prevalence increases with age.^1^ As in the literature, we found a higher prevalence of index cases in adult males; only 8% of cases were aged over 65 years.

The T102C polymorphism appears to be unrelated with OSAS, as the control group had the same frequency as index cases for the TT/TC and CC genotypes; this finding concurs with other published results.^8^, ^9^ Although the 5-HT2A receptor is mostly related with serotonin (5-HT) excitation within the representative motor nucleus of upper airways, lack of common polymorphisms or epigenetic changes that alter messenger RNA levels for the *5-HTR2A* gene may explain the absence of association between the T102C polymorphism and OSAS in this population; it is also considered a silent polymorphism and was present in a similar proportion in the control group.^8^, ^9^, ^13^, ^14^

The genotype AA was significantly higher in index cases, whereas the genotypes GG/GA were higher in controls, meaning that relative to the -1438G/A polymorphism, the genotype AA may be associated with OSAS in the study population; this finding concurs with other published results.^8^, ^9^

There was a significant gender difference between index cases and controls in relation to the -1438G/A polymorphism genotypes, which was not the case with T102C polymorphism genotypes. The comparison of genotypes with gender was done because of a higher prevalence of OSAS in males, as shown in the literature.^1^, ^6^, ^9^, ^19^

There was no correlation between the AHI (obtained during polysomnography) and the genotypes of both polymorphisms in index cases, which again concurs with the literature.^8^, ^9^ This condition may suggest that other mechanisms besides *5-HTR2A* gene polymorphisms may be involved in the pathophysiology of OSAS. It is evident that OSAS is a phenotype of several related or unrelated disorders, and that it results from a complex association between genes and environmental modifying factors.^9^ This may explain the statistically significant absence of association between both polymorphisms and the polysomnographic findings in the present study.

Airway size variations are probably defined by genetic influences on bone structure, and tongue and tonsil size; there may also be other factors that are acquired because of obesity.^13^, ^20^, ^21^, ^22^ Although *5-HTR2A* gene polymorphisms may have an important role in the activity of pharyngeal dilating muscles, other factors such as cephalometric characteristics and soft tissue structures may be determinant for opening the airways during sleep.^8^, ^23^, ^24^ Absence of craniofacial dimorphism and temporomandibular alterations and a BMI ≤ 35 kg/m^2^ were two of the inclusion criteria for this study. These criteria were applied strictly to exclude these variables - and others - which affect upper airway patency. Thus, when the BMI was compared with the genotypes of both polymorphisms in index cases and controls, the BMI from 25 to 35 kg/m^2^ was related significantly with the -1438G/A polymorphism genotypes in index cases; this result concurs with those of the literature,^8^, ^9^, ^25^ as it results from more cases in this BMI range rather than the effect of the polymorphism.

Therefore, the T102C polymorphism appears to be unrelated with OSAS in our sample, compared with the -1438G/A polymorphism. This in turn may be associated with OSAS in the study population, especially in males with a BMI from 25 to 35 kg/m^2^, since the prevalence was higher among patients presenting these variables.

Genetic studies are being carried out to clarify some of the complexities of sleep and to open new therapeutic approaches for treating sleep disorders.^25^, ^26^, ^27^ Genes are probably les involved in changes of state (non-REM->REM) that are seen electrophysiologically during the sleep cycle; rather, genes are certainly related with the circadian rhythm and sleep homeostasis.^28^ The most common sleep disorders result from the interaction of several genes and environmental factors. Understanding the impact of genetic factors will help elucidate the pathophysiology of sleep disorders, and may provide support for therapeutic approaches.^25^, ^26^, ^27^

## CONCLUSION

Using molecular techniques (PCR/RFLP) we identified T102C and -1438G polymorphisms of the *5-HTR2A* gene, thereby adding to the molecular investigation of the OSAS. Serotonergic mechanisms may be related with the OSAS. There were no differences in the prevalence of the T102C polymorphism among OSAS patients and controls. There is evidence that the -1438G/A polymorphism is associated with the OSAS.
